# Vertical distribution of *Phytophthora agathidicida* oospore DNA in kauri forest soils: implications for optimized sampling and disease monitoring

**DOI:** 10.1128/spectrum.01025-26

**Published:** 2026-07-29

**Authors:** Jade T. T. Palmer, Emily M. G. Hocking, Monica L. Gerth

**Affiliations:** 1 School of Biological Sciences, Victoria University of Wellington 8491, Wellington, Aotearoa, New Zealand; USDA-ARS San Joaquin Valley Agricultural Sciences Center, Parlier, California, USA

**Keywords:** kauri dieback, soil sampling, oospore detection, quantitative PCR, diagnostic assays, oDNA

## Abstract

*Phytophthora* species are globally significant soilborne oomycetes responsible for widespread ecosystem decline. Standard soil sampling protocols, originally developed for qualitative baiting assays, typically require collecting substantial soil volumes to capture viable propagules. While effective for culture-based detection, these protocols are labor-intensive and can damage the shallow root systems of sensitive host species such as New Zealand kauri (*Agathis australis*). *Phytophthora agathidicida* (PA), the causal agent of kauri dieback, is routinely surveyed using these methods. However, quantitative data describing the vertical distribution of PA in natural forest soils are lacking. Consequently, it remains unclear where in the soil profile PA is most concentrated and, therefore, the depth that should be sampled to ensure optimal detection. In this study, we applied a quantitative oospore DNA (oDNA) qPCR assay to characterize the fine-scale vertical distribution of PA at four soil depth increments (0–5, 6–10, 11–15, and 16–20 cm) from 12 kauri trees representing a range of disease stages. Results revealed distinct vertical stratification, with PA DNA concentrations peaking within the upper 0–10 cm of soil in non-symptomatic and possibly symptomatic trees. In symptomatic trees, the absolute peak occasionally reached 11–15 cm, while pathogen signals remained consistently detectable within the top 10 cm. Field validation from an additional eight trees confirmed that targeted 0–10 cm “shallow” sampling yielded higher PA concentrations than deeper sampling. Together, these findings provide a quantitative basis for refining soil sampling strategies, which will enable more sensitive molecular detection while minimizing disturbance and logistical effort in fragile ecosystems.

**IMPORTANCE:**

*Phytophthora* species are among the most destructive soilborne pathogens globally, requiring robust diagnostic tools for both agricultural and conservation settings. Traditional sampling methods were designed around the biological requirements of baiting bioassays, which often necessitate collecting large soil volumes over broad depth profiles to maximize the likelihood of recovering viable propagules. However, this high-volume collection is labor-intensive and can cause significant soil and root disturbance in sensitive forest ecosystems. This study provides a quantitative assessment of how *Phytophthora agathidicida* (PA) DNA is distributed vertically in the soil profile around kauri trees at different stages of disease severity. We demonstrated that while peak absolute abundance shifts within the 0–15 cm range as infection progresses, the pathogen signal remains consistently detectable within the top 10 cm. Our findings indicate that targeted, shallow sampling enhances sensitivity by reducing signal dilution, thereby offering a lower-impact pathway for kauri dieback surveillance.

## INTRODUCTION

*Phytophthora* species are among the most destructive soilborne oomycetes worldwide, causing severe agricultural losses and widespread ecosystem decline (1–4). Traditional surveillance methods have been standardized around the requirements of baiting methods, which typically require large soil volumes collected from broad depth profiles to maximize pathogen recovery (5, 6). This high-volume sampling approach is particularly problematic in New Zealand kauri (*Agathis australis*) forests, where *Phytophthora agathidicida* (PA) causes widespread dieback (7–9). Because kauri trees possess extensive, highly sensitive fine-root systems concentrated in surface soils, the excavation of large soil volumes is strongly discouraged (10). However, sampling protocols for PA have recommended collecting large, composite soil samples comprising subsamples taken from several points (typically 4–8) around a tree, to a depth of approximately 15–20 cm, yielding a total soil volume of roughly 1 kg per sample (11, 12). These methods are labor-intensive, disturb fine kauri roots, and require considerable effort for sample storage, transport, and disposal.

Currently, the empirical evidence underpinning PA soil sampling protocols is limited. One study examined soil from two symptomatic kauri trees using baiting to assess the depth distribution (13). Detection was successful to depths of 14 cm for one tree and 20 cm for the second (13). However, the small sample size and the qualitative nature of baiting mean that little is known about the abundance of *P. agathidicida* beyond its potential presence in the upper 0–20 cm. Studies of other *Phytophthora* species generally report the highest recovery from upper soil layers (0–10 cm), though results vary among species and depend on factors such as host root distribution and environmental context (e.g., agricultural fields, forests, and nurseries) (14–17). Furthermore, the vertical distribution of *Phytophthora* can extend significantly deeper in soil; for example, *P. cinnamomi* has been recovered from depths down to 74 cm (18).

The development of a filtration-based oospore DNA (oDNA) enrichment method coupled with quantitative PCR (qPCR) for *P. agathidicida* has enabled pathogen abundance to be measured from as little as 10–15 g of soil (19, 20). This targeted molecular approach reduces the need for large soil volumes and provides higher detection rates in known positive field samples than total DNA extracted from soil (19, 21). However, this shift toward smaller sample volumes necessitates a re-evaluation of the optimal sampling depth. Here, we present the first quantitative assessment of *P. agathidicida* oospore DNA distribution at different soil depths in kauri forests. We examined 20 trees across two sites, using detailed soil coring for 12 trees at one site and a simpler shallow-versus-deep sampling design for eight trees at the other. Combined, these data inform evidence-based recommendations for revised soil sampling protocols and demonstrate how quantitative molecular approaches may be used to improve *Phytophthora* diagnostics and monitoring in sensitive forest ecosystems.

## MATERIALS AND METHODS

### Soil core sampling: Detailed depth profile analysis

Soil cores were collected from 12 individual kauri trees (labeled KM1–KM12) at Kauri Mountain, Whangārei Heads, Northland, New Zealand. These trees included three symptom severity categories (i.e., non-symptomatic, possibly symptomatic, and symptomatic) to capture potential variation in pathogen load. The symptom category was scored in the field by an experienced observer using standard visual assessment criteria (i.e*.,* canopy condition and presence of lesions) (22).

At each tree, four soil cores (19 mm in diameter and 20 cm in length) were collected at approximately 1 m distances from the trunk in the uphill, downhill, left, and right directions and 90° apart. Each core was subdivided into four depth segments: 0–5 cm, 6–10 cm, 11–15 cm, and 16–20 cm. Sampling points were adjusted as necessary to avoid roots, rocks, or other obstructions, and alternative cores were taken within 30 cm of the original sampling location if the target depth could not be reached. When the upper 10 cm was too loose or fibrous for core sampling, the 0–5 cm and 6–10 cm layers were collected with a trowel, and the corer was used for the lower sections. All sampling equipment was cleaned with bleach wipes between each core and twice between trees to minimize the risk of cross-contamination. Soil samples were stored at 4 °C until extraction, and all samples were processed within 7 days of collection. Soil collected at each depth for each tree was pooled and hand-homogenized, yielding four composite samples per tree and 48 samples in total. All sample processing was conducted aseptically in a laminar-flow hood, and laboratory surfaces and equipment were regularly decontaminated with bleach and a DNase-based reagent throughout the extraction procedures.

### Trowel-Based soil sampling: Comparing depths in field conditions

In addition to core sampling, trowel-based sampling was conducted under realistic field conditions to compare two depths: approximately 0–10 cm (“shallow”) or 0–20 cm (“deep”). These soil samples were collected from Waipoua Forest, Northland, New Zealand, by the Kauri Ora Rangers of Te Roroa Iwi. Eight soil samples were collected from around eight individual kauri trees (labeled TR1–TR8); we requested that they sample trees with visible symptoms for this comparison. Samples at each depth were combined and homogenized in the lab into one composite sample per tree. This produced 16 composite samples total (eight shallow and eight deep) for oDNA-qPCR analysis.

### DNA extraction and storage

All 64 soil samples were processed using the oDNA method as previously described (19, 20). The only modification to the original protocol was in the final elution and storage steps. DNA was eluted in a low-EDTA TE buffer (10 mM Tris-HCl and 0.1 mM EDTA) rather than the manufacturer’s supplied buffer to ensure greater DNA stability during storage. Extracted DNA was stored at −20°C in DNA LoBind tubes (Eppendorf), which helped reduce DNA adhesion to tube walls, thereby maximizing DNA recovery for quantitative PCR (qPCR) and preserving sample integrity for potential downstream analyses.

### Quantitative PCR (qPCR)

All qPCR assays were performed using a QuantStudio 3 Real-Time PCR System (Applied Biosystems). Each assay employed fluorescein amidite (FAM; Macrogen) as the fluorescent dye and Black Hole Quencher 1 (BHQ1; Macrogen) as the quencher. The PA qPCR conditions were as follows: an initial denaturation at 95°C for 10 min, followed by 40 cycles of 95°C for 15 s, and 58°C for 60 s. Each qPCR was set up in a 30μL final volume containing 1× HOT FIREPol Probe Universal qPCR Mix, 400 nM of each primer, 250 nM of the TaqMan probe, and 3 μL of input DNA. The primers and probe used for quantification of PA were as follows: PA-LTR-for 5ʹ-ACGCGCTCTGTTTCTTTAGC-3ʹ; PA-LTR-rev 5ʹ-GCGGGCTTCCATTCAATTCA-3ʹ; and PA-LTR-TaqMan probe, 5′-FAM-TAGTATGCGCTTTTGAGGAAGCGTAA-BHQ1-3′ (19, 20). The genus-level *Phytophthora* qPCR conditions were as follows: an initial denaturation at 95°C for 10 min, followed by 40 cycles of 95°C for 15 s, and 60°C for 60 s. Each qPCR was set up in a 30μL final volume containing 1× HOT FIREPol Probe Universal qPCR Mix, 400 nM of each primer, 250 nM of the TaqMan probe, and 3 μL of input DNA. The primers and probe used for genus-level quantification of *Phytophthora* spp. were as follows: PhyG-F2 5′-CGTGGGAATCATAATCCT-3′ and PhyG-Rb 5′-CAGATTATGAGCCTGATAAG-3′, along with the probe TrnM_PhyG_probe2 5′-ATRTTGTAGGTTCAARTCCTAYCATCAT-3′ (23).

For standard curve generation, genomic DNA was extracted from *P. agathidicida* isolate 3770. Mycelial mats were grown in the dark at 22°C in potato-dextrose broth (Difco) until they covered approximately 50% of the 90-mm Petri dish surface. The mats were harvested, washed with sterile water, dried using Kimwipes, and ground to a fine powder in liquid nitrogen using a mortar and pestle. DNA was extracted using the DNeasy Plant Mini Kit (Qiagen) according to the manufacturer’s protocol. DNA concentrations were quantified using the Qubit dsDNA High-Sensitivity Assay kit.

Ten genomic DNA standards in a tenfold dilution series from 0.03 fg to 30,000,000 fg (30 ng) were prepared in DNA LoBind tubes (Eppendorf), with nine replicates per concentration. Cycle threshold (CT) values were plotted against log_10_-transformed DNA concentrations to construct the standard curve and calculate efficiency and R^2^ (Fig. S1A). The limits of detection and quantification were determined using previously developed algorithms (24) (Fig. S1B). Seven genomic DNA standards in a twofold dilution series from 0.16 fg to 60 fg were prepared in DNA LoBind tubes, with nine replicates per concentration. The coefficient of variation for the limit of quantification was set to 0.35, and both algorithms were set to “Best,” as recommended (24). The calculated limit of detection (LoD) was 0.7 fg, and the limit of quantification (LoQ) was 1 fg.

Subsequent qPCR experiments used seven standards from the most linear range of the standard curve, from 3 fg to 3,000,000 fg (3 ng), to determine the concentration of PA DNA in soil samples. All reactions were performed in triplicate, and final concentrations are expressed as fg of PA DNA per gram of soil (fg/g), accounting for the total elution volume of the DNA extract and the initial fresh soil mass. Negative controls (distilled water) and a spiked inhibition control (3 fg spike of PA genomic DNA) were included in each run to monitor for contamination and potential PCR inhibition.

## RESULTS

### Relationships between *P. agathidicida* abundance, depth, and symptom status

**Fig 1:**
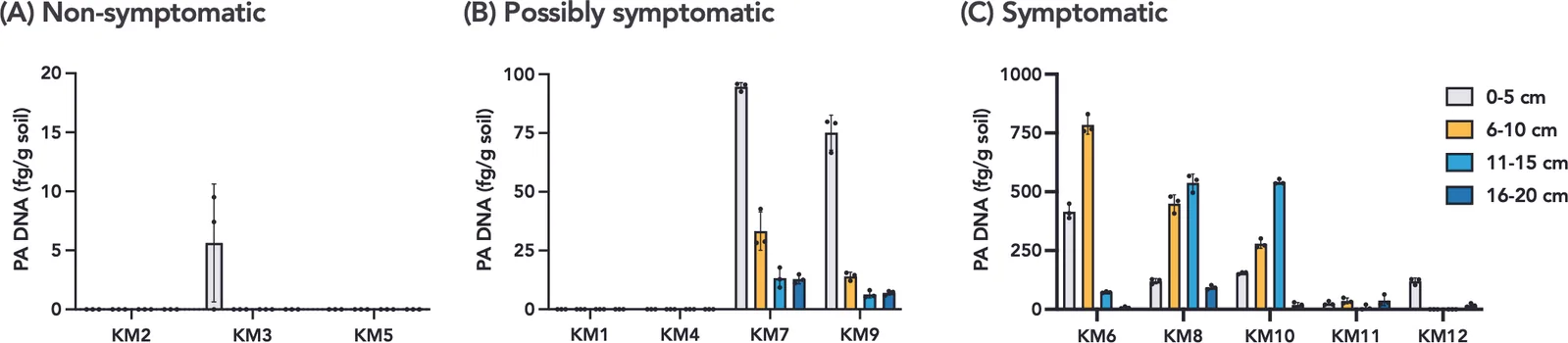
Distribution of *Phytophthora agathidicida* (PA) DNA at different depths in soil adjacent to individual kauri trees with different degrees of symptom severity. Because PA DNA loads differed markedly among symptom categories, the y-axis scales differ among panels **A**, **B**, and **C**. Soil cores were collected adjacent to kauri trees classified as (**A**) non-symptomatic, (**B**) possibly symptomatic, or (**C**) symptomatic, and cores were sectioned into four depth intervals (0–5, 6–10, 11–15, and 16–20 cm; color-coded as indicated in the key). Concentrations of PA DNA were quantified by qPCR and are expressed as fg PA DNA per g soil: mean ± standard deviation (*n* = 3) for each tree (sample IDs KM1 to KM12).

Twelve kauri trees representing three symptom severity categories were sampled at four depth intervals (0 to 20 cm), yielding 48 total samples (Fig. 1). Of these 12 trees, eight tested positive for PA DNA using the oDNA-qPCR method. The mean PA DNA concentration peaked at 200 fg/g soil in the 6- to 10-cm layer, before declining to a minimum of 25 fg/g soil in the deepest layer sampled (16–20 cm) (Table S1). Differences among these depths were not statistically significant, reflecting the substantial variation among individual trees, with some showing high concentrations and others showing little or no detectable PA DNA. The depth profiles were then examined on a tree-by-tree basis and considered in relation to the symptom status. Detailed statistical analysis of these profiles is provided in Table S2.

Non-symptomatic trees (*n* = 3): PA DNA was undetectable in soil around two trees (KM2 and KM5). Soil around the third tree (KM3) showed only low levels of PA DNA (~5 fg/g soil), which was restricted to the surface layer (0 to 5 cm).

Possibly symptomatic trees (*n* = 4): PA DNA was detected in soil around two of the four trees, predominantly in the 0- to 5-cm layer and at moderate levels (~75–100 fg/g soil), with concentrations declining rapidly with depth.

Symptomatic trees (*n* = 5): PA was detected in the soil around all five symptomatic trees. These trees exhibited the broadest vertical spread of PA DNA. Elevated levels (~100–800 fg/g soil) were typically detected in the top three layers (0–5, 6–10, and 11–15 cm), with markedly lower amounts at 15–20 cm.

### Vertical distribution of *Phytophthora* spp. DNA

**Fig 2:**
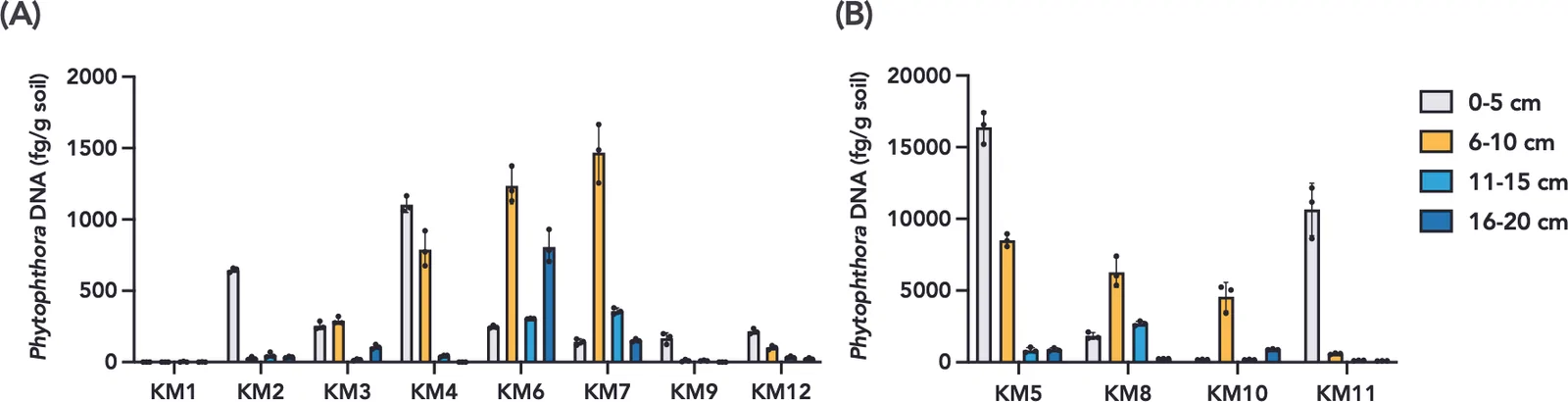
Distribution of *Phytophthora* spp. DNA at different soil depths. Because *Phytophthora* spp. DNA loads differed markedly among the sampled trees, the y-axis scales differ by 10-fold between panels **A** and **B**. Soil cores were collected adjacent to 12 kauri trees and sectioned into four depth segments (0–5, 6–10, 11–15, and 16–20 cm; color-coded as indicated in the key). Concentrations of *Phytophthora* spp. DNA were quantified by qPCR and are expressed as fg of DNA per g soil (mean ± standard deviation of three technical replicates) for each tree (sample IDs KM1 to KM12).

For comparison, the vertical distribution of *Phytophthora* spp. DNA was quantified using genus-level primers and the same depth profiles (Fig. 2 and statistical details provided in Table S3). While total concentrations varied significantly among individual trees, ranging from undetectable levels to >16,000 fg/g, the depth-related pattern was highly consistent. In 11 of the 12 trees sampled, the highest abundance of *Phytophthora* spp. DNA occurred in one of the two upper soil layers (0–5 cm or 6–10 cm; *P* < 0.0001), regardless of the symptom severity category or total *Phytophthora* spp. abundance. The sole exception was KM1, which had comparatively low levels in the two deepest soil layers (11–20 cm); however, this profile did not differ significantly among depths (*F* [3, 8] = 0.8669, *P* = 0.4969). Given that these concentrations represented only a small fraction of the total site-wide *Phytophthora* spp. DNA abundance, KM1 did not alter the overall trend. Collectively, these profiles reinforce that shallow sampling (0–10 cm) is optimal for detecting the most *Phytophthora* DNA in kauri forest soils.

### Comparison of standard and modified shallow sampling protocols

The detailed depth profiles from the soil cores suggested that shallower sampling could effectively capture PA when present. To evaluate whether this finding could translate into routine field sampling, soil samples were collected around eight kauri trees considered potentially symptomatic. Two sampling methods were compared: the standard protocol, which combines soil from eight holes dug to approximately 20 cm depth into a single composite (“deep”) sample, and a modified “shallow” sampling approach, which collected soil from eight nearby holes dug only to about 10 cm depth.

**Fig 3:**
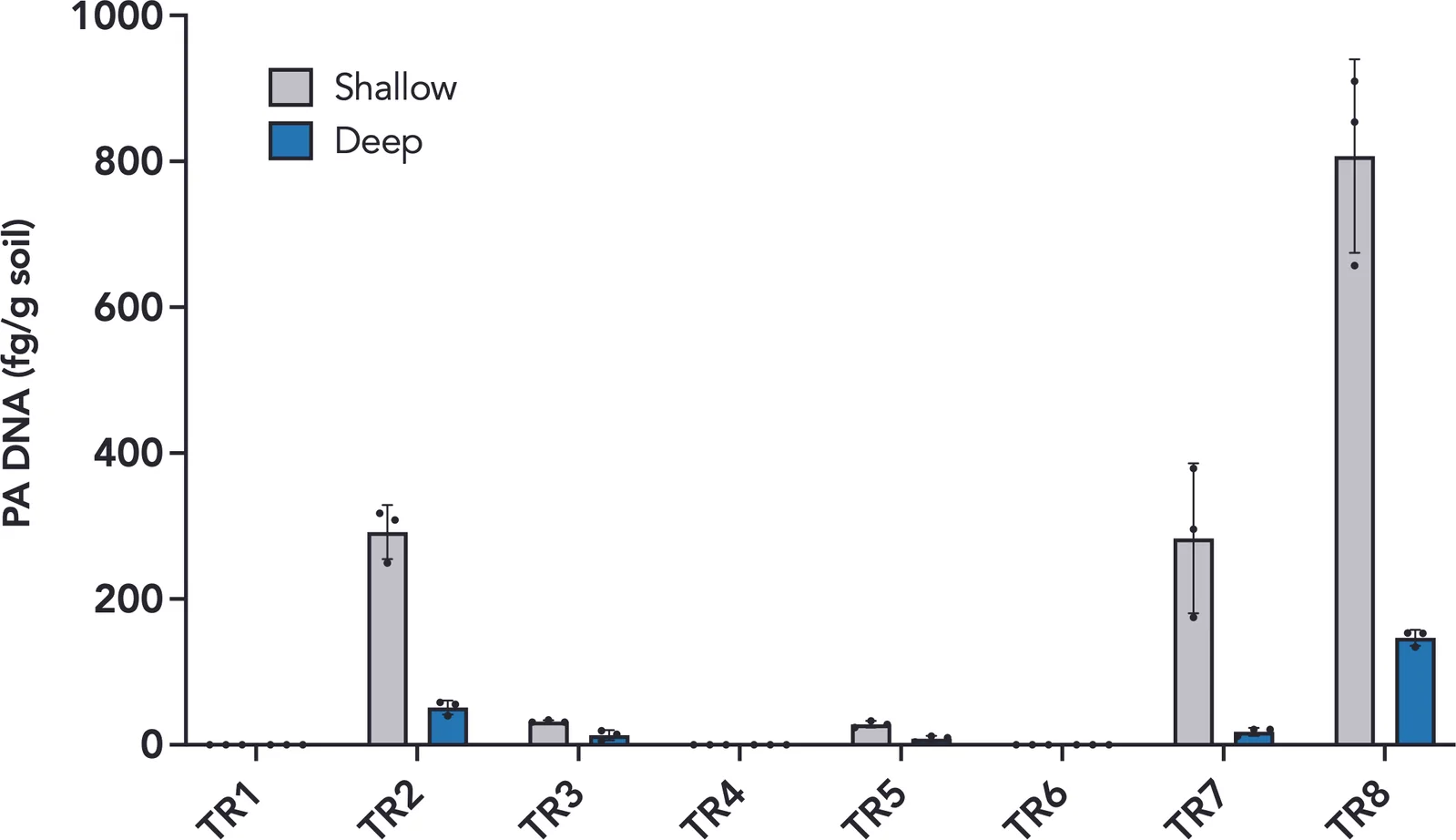
Quantification of Phytophthora agathidicida (PA) DNA at two soil sampling depths adjacent to kauri trees. Soil samples were collected at two depths adjacent to trees classified as possibly symptomatic: approximately 0–10 cm, shallow, and 0–20 cm, deep (colour-coded as indicated in the key). PA DNA concentrations were quantified by qPCR and are expressed as fg of PA DNA per g of soil (mean ± range of three technical replicates) around each tree (Tree IDs TR1–TR8). PA DNA was not detected at either depth for trees TR1, TR4, and TR6.

Of the eight trees sampled, five tested positive for PA using both approaches; PA was not detected in the samples from TR1, TR4, and TR6. For the five positive trees, the “shallow” soil samples contained higher concentrations of PA DNA than the corresponding “deep” samples (Fig. 3). When evaluated by individual tree, PA DNA concentrations were significantly lower in the deep soil samples compared to the shallow soil samples for the soil surrounding every positive kauri tree (Welch's t-test, *P* < 0.05; see Table S4 for full statistical details).

## DISCUSSION

To our knowledge, this is the first study to quantify PA oospore DNA in soil at fine-scale depths around individual kauri trees. Our results reveal a distinct vertical stratification: the highest PA concentrations occurred in the upper 0- to 5-cm segment around non-symptomatic and possibly symptomatic trees and then shifted to the 6- to 15-cm segments around trees with more prominent symptoms. Because these patterns were consistently detectable within the top 10 cm associated with all positive trees, our data indicate that shallower sampling maximizes the detection. In contrast, standard protocols that pool soil from 0 to 20 cm risk diluting pathogen signals, thereby reducing diagnostic performance and possibly detection.

In a trial using eight trees, smaller, shallower cores (0–10 cm) yielded significantly higher PA concentrations than standard deeper protocols, confirming improved sensitivity with reduced soil volumes. This consistency indicates that revising standard operating procedures to prioritize sampling at 0–10 cm could enhance PA oDNA detection efficiency. These abundance profiles suggest that shallower sampling may also benefit soil baiting or other DNA-based detection methods by targeting the depth at which inocula are most concentrated.

**Fig 4:**
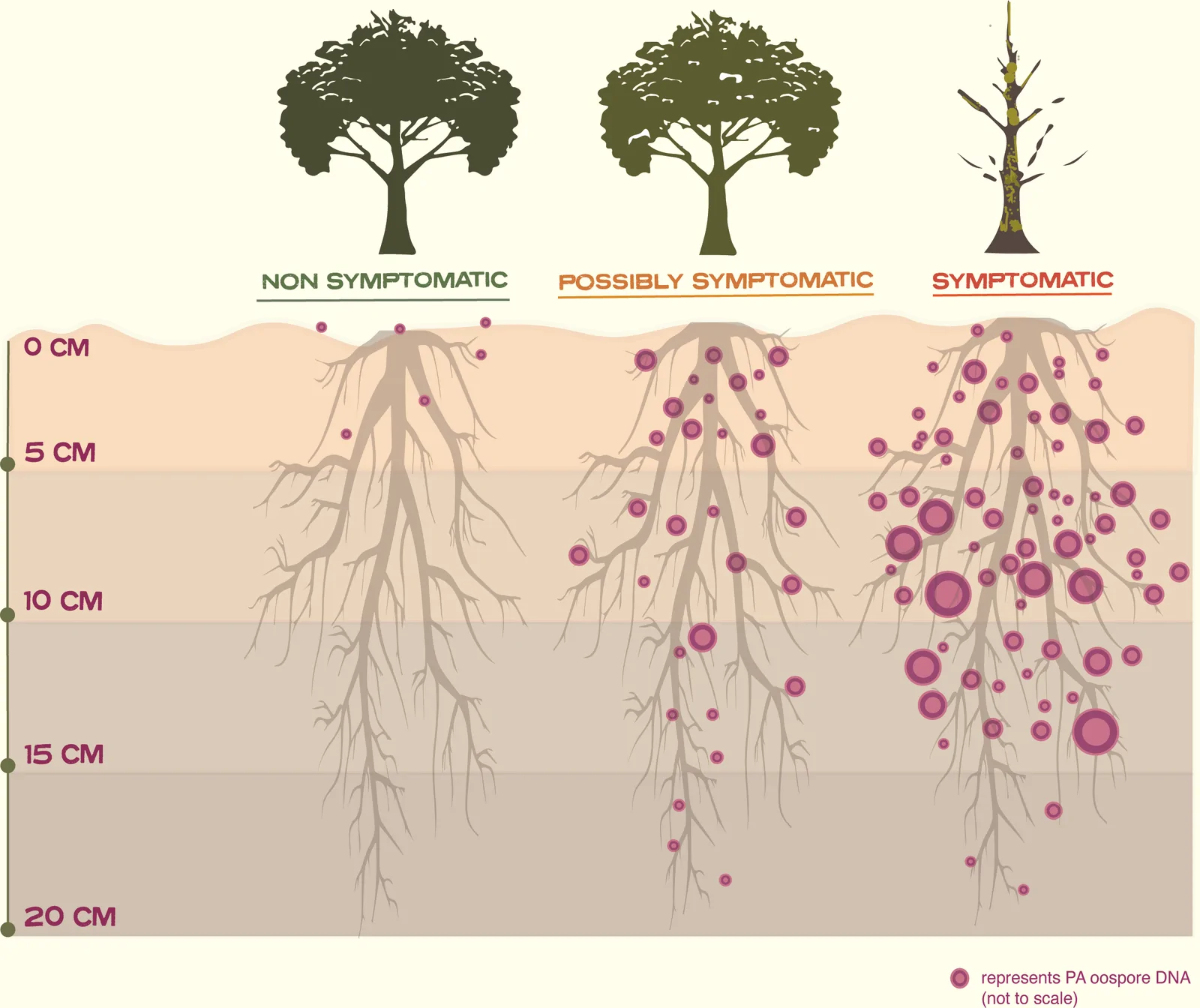
Schematic model of vertical distribution patterns of oospore DNA from *Phytophthora agathidicida* (PA). This conceptual diagram illustrates general trends: PA DNA is typically shallowest in soil around non-symptomatic trees, more widely distributed in soil around possibly symptomatic trees, and peaks deeper in the soil around symptomatic trees while remaining detectable in upper soil layers. Note: Relative scales and patterns are illustrative only.

While representing a single temporal snapshot, the quantitative data from trees at various stages of disease severity provide insights into disease dynamics. Our data support the model shown in Fig. 4, where soils around apparently healthy trees may contain low levels of PA (<5 fg/g soil) at shallow depths. These low levels likely reflect introduction via contaminated footwear, equipment, animal activity, or water movement. As infection progresses, pathogen reproduction and oospore production within host tissues increase PA DNA concentrations, with early-stage or possibly symptomatic trees yielding up to ~100 fg/g soil. In later stages of disease development, root dieback releases oospores into the surrounding soil; soil around severely affected or dying trees generally contains more than 100 fg/g soil of PA DNA.

These concentration thresholds provide a quantitative benchmark for relating pathogen load to field observations. Longitudinal monitoring of trees with low and intermediate oospore loads will be important for refining these disease dynamics over time. For example, we have observed apparently healthy kauri trees (e.g., KM3) in this study and at other sites (unpublished data) with low PA DNA levels (~5 fg/g soil or less). Tracking these at-risk trees could reveal the PA concentration associated with symptom development. Similarly, quantitative monitoring of trees with intermediate oospore loads (e.g*.*, KM7 and KM9) could provide insights into when oospore production peaks during the disease cycle, while tracking soil from around dead trees could determine PA persistence in soils.

Our oDNA assay facilitates this transition from qualitative to quantitative assessment and offers several methodological advantages over both baiting and broad environmental DNA (eDNA) or metagenomic methods for PA detection. Unlike baiting, which depends on propagule germination and can be confounded by environmental dormancy or microbial competition (25, 26), the oDNA assay directly targets pathogen DNA, providing a quantitative measure that is independent of these physiological constraints. While standard eDNA analyses capture a complex mixture of intracellular and extracellular DNA, the oDNA protocol employs size-selection filtration to enrich for oospore-sized particles, reducing the likelihood that results are skewed by relic DNA. Because oospores are thick-walled survival structures, they are expected to persist in soil (8, 27), although the assay does not distinguish viable from non-viable spores. Ultimately, the assay provides a rapid, quantitative assessment of the oospore load in soil from minimal sample volumes.

The methodology presented here is also potentially adaptable to a wide range of research questions on *Phytophthora* where pathogen load is a key factor, such as mapping horizontal spread, evaluating containment strategies, or assessing the effectiveness of chemical treatments. By moving from qualitative presence/absence to high-resolution quantification, we can better understand and manage the small-scale pathogen dynamics that drive large-scale forest decline.

## SUPPLEMENTAL MATERIAL

Supplemental Material spectrum.01025-26-s0001.pdf Fig. S1; Tables S1 to S4.

ASM does not own the copyrights to Supplemental Material that may be linked to, or accessed through, an article. The authors have granted ASM a non-exclusive, world-wide license to publish the Supplemental Material files. Please contact the corresponding author directly for reuse.

## References

[1] Kamoun S, Furzer O, Jones JDG, Judelson HS, Ali GS, Dalio RJD, Roy SG, Schena L, Zambounis A, Panabières F, et al.. 2015. The top 10 oomycete pathogens in molecular plant pathology. Mol Plant Pathol 16:413–434. doi:10.1111/mpp.12190 25178392 PMC6638381

[2] Jung T, Pérez-Sierra A, Durán A, Horta Jung M, Balci Y, Scanu B. 2018. Canker and decline diseases caused by soil- and airborne *Phytophthora* species in forests and woodlands. Persoonia 40:182–220. doi:10.3767/persoonia.2018.40.08 30505001 PMC6146643

[3] Scott P, Bader MKF, Burgess T, Hardy G, Williams N. 2019. Global biogeography and invasion risk of the plant pathogen genus *Phytophthora*. Environmental Science & Policy 101:175–182. doi:10.1016/j.envsci.2019.08.020

[4] Fones HN, Bebber DP, Chaloner TM, Kay WT, Steinberg G, Gurr SJ. 2020. Threats to global food security from emerging fungal and oomycete crop pathogens. Nat Food 1:332–342. doi:10.1038/s43016-020-0075-0 37128085

[5] Burgess TI, López‐Villamor A, Paap T, Williams B, Belhaj R, Crone M, Dunstan W, Howard K, Hardy GE St J. 2021. Towards a best practice methodology for the detection of *Phytophthora* species in soils. Plant Pathol 70:604–614. doi:10.1111/ppa.13312

[6] Tiakina Kauri. 2023. Approved soil and root sampling method for Phytophthora agathidicida detection. Available from: https://www.kauriprotection.co.nz/assets/content-blocks/downloads/Tiakina-Kauri-1-SOP-for-Soil-and-root-Sampling-final-June-2023.pdf

[7] Weir BS, Paderes EP, Anand N, Uchida JY, Pennycook SR, Bellgard SE, Beever RE. 2015. A taxonomic revision of *Phytophthora* Clade 5 including two new species, *Phytophthora agathidicida* and *P. cocois*. Phytotaxa 205:21–38. doi:10.11646/phytotaxa.205.1.2

[8] Bradshaw RE, Bellgard SE, Black A, Burns BR, Gerth ML, McDougal RL, Scott PM, Waipara NW, Weir BS, Williams NM, Winkworth RC, Ashcroft T, Bradley EL, Dijkwel PP, Guo Y, Lacey RF, Mesarich CH, Panda P, Horner IJ. 2020. *Phytophthora agathidicida*: research progress, cultural perspectives and knowledge gaps in the control and management of kauri dieback in New Zealand. Plant Pathol 69:3–16. doi:10.1111/ppa.13104

[9] Mainwaring JC, Vink JNA, Gerth ML. 2023. Plant-pathogen management in a native forest ecosystem. Curr Biol 33:R500–R505. doi:10.1016/j.cub.2023.02.047 37279683

[10] Tiakina Kauri. 2025. Best practice guide: kauri ora: hygiene principles Available from: https://www.kauriprotection.co.nz/assets/Documents-PDFs/Best-Practice-Guides/Kauri-Ora-Hygiene-Principles-guide.pdf

[11] Bellgard SE, Dick MA, Horner IJ. 2011. Analysis of kauri dieback soil samples, Phase 1 Final report. Prepared for the Ministry of Agriculture and Forestry. Contract RFQ12239. Available from: https://www.kauriprotection.co.nz/assets/Research-reports/Surveillance-Detection-Diagnostics-and-Pathways/Analysis-of-Kauri-Dieback-soil-and-tissue-samples/ANALYS1.PDF

[12] Tiakina Kauri. 2023. Approved soil baiting method for *Phytophthora agathidicida*. Ministry for Primary Industries. https://www.kauriprotection.co.nz/assets/content-blocks/downloads/MPI-Approved-Test-Soil-bioassay-baiting-protocol-FINAL-June-2023.pdf.

[13] Bellgard SEW, Bevan S, Pennycook SR Paderes E Beever RE, Than DJ DJ, Hill L, Williams SE. 2013. Specialist Phytophthora research: biology, pathology, ecology and detection of PTA: MPI contract 11927. Ministry for Primary Industries, Wellington, New Zealand7 Available from: https://www.kauriprotection.co.nz/assets/Research-reports/Understanding-the-disease/Specialist-Phytophthora-Research-Biology-Pathology-Ecology-and-Detection-of-PTA.pdf

[14] Horner IJ, Wilcox WF. 1996. Spatial distribution of *Phytophthora cactorum* in New York apple orchard soils . Phytopathology 86:1122. doi:10.1094/Phyto-86-1122

[15] Ristaino JB, Gumpertz ML. 2000. New frontiers in the study of dispersal and spatial analysis of epidemics caused by species in the genus *Phytophthora*. Annu Rev Phytopathol 38:541–576. doi:10.1146/annurev.phyto.38.1.541 11701854

[16] Dart NL, Chastagner GA, Rugarber EF, Riley KL. 2007. Recovery frequency of *Phytophthora ramorum* and other *Phytophthora spp*. in the soil profile of ornamental retail nurseries. Plant Dis 91:1419–1422. doi:10.1094/PDIS-91-11-1419 30780757

[17] Kaur Y, Arora A, Thind SK. 2022. Spatial distribution of *Phytophthora nicotianae* in citrus rhizosphere. Indian Phytopathology 75:969–977. doi:10.1007/s42360-022-00553-1

[18] Meadows IM, Jeffers SN. 2011. Distribution and recovery of *Phytophthora* cinnamomi in soils of mixed hardwood-pine forests of the south-eastern USA. New Zealand Journal of Forestry Science 41S:S39–S47.

[19] Palmer JTT, Vink JNA, Castro LM, Craig OJS, Davison EE, Gerth ML. 2025. Improved isolation and PCR detection of *Phytophthora agathidicida* oospores from soils. Microbiol Spectr 13:e0013525. doi:10.1128/spectrum.00135-25 40197128 PMC12073851

[20] Palmer JTT, Gerth ML. 2025. A method for the separation of *Phytophthora* oospores from soil for DNA-based detection. Methods Mol Biol 2892:139–149. doi:10.1007/978-1-0716-4330-3_10 39729274

[21] McDougal R, Bellgard S, Scott P, Ganley B. 2014. Comparison of a real-time PCR assay and a soil bioassay technique for detection of Phytophthora Taxon Agathis from soil. Report 53789. New Zealand Forest Research Limited. Available from: https://www.kauriprotection.co.nz/assets/Research-reports/Surveillance-Detection-Diagnostics-and-Pathways/Comparison-of-a-real-time-PCR-assay-soil-bioassay-PA.pdf

[22] Dick MA, Bellgard SE. 2010. Preliminary survey for *Phytophthora Taxon* Agathis. Ministry of Agriculture and Forestry. Report 46320. https://www.kauriprotection.co.nz/assets/Research-reports/Surveillance-Detection-Diagnostics-and-Pathways/Preliminary-survey-for-Phytophthora.pdf.

[23] Bilodeau GJ, Martin FN, Coffey MD, Blomquist CL. 2014. Development of a multiplex assay for genus- and species-specific detection of *Phytophthora* based on differences in mitochondrial gene order. Phytopathology 104:733–748. doi:10.1094/PHYTO-09-13-0263-R 24915428

[24] Klymus KE, Merkes CM, Allison MJ, Goldberg CS, Helbing CC, Hunter ME, Jackson CA, Lance RF, Mangan AM, Monroe EM, Piaggio AJ, Stokdyk JP, Wilson CC, Richter CA. 2020. Reporting the limits of detection and quantification for environmental DNA assays. Environmental DNA 2:271–282. doi:10.1002/edn3.29

[25] Sarker SR, Burgess TI, Hardy GEStJ, McComb J. 2023. Closing the gap between the number of *Phytophthora species* isolated through baiting a soil sample and the number revealed through metabarcoding. Mycol Progress 22:39. doi:10.1007/s11557-023-01892-7

[26] Bačová A, Cooke DEL, Milenković I, Májek T, Nagy ZÁ, Corcobado T, Randall E, Keillor B, Cock PJA, Jung MH, Jung T, Tomšovský M. 2024. Hidden *Phytophthora* diversity unveiled in tree nurseries of the czech republic with traditional and metabarcoding techniques. Eur J Plant Pathol 170:131–156. doi:10.1007/s10658-024-02886-1

[27] Horner IJ, Hough E. 2015. Assay of stored soils for presence of Phytophthora agathidicida. A Plant and Food Research report prepared for The Ministry for Primary Industries Contract No. 32294. Available from: https://www.kauriprotection.co.nz/assets/Research-reports/Understanding-the-disease/Assay-of-stored-soils-for-presence-of-Phytophthora-agathidicida.pdf

