# Supplementary material for: Vertical distribution of Phytophthora agathidicida oospore DNA in kauri forest soils: implications for optimized sampling and disease monitoring
Source: Microbiol Spectr. 2026 Jul 29;14(9):e01025-26. doi: 10.1128/spectrum.01025-26 (PMC13532275; doi:10.1128/spectrum.01025-26)
Supplement: Supplementary file 1 [file spectrum.01025-26-s0001.pdf]

Supplementary Information

Vertical distribution of *Phytophthora agathidicida* oospore DNA in kauri forest soils: Implications for optimised sampling and disease monitoring

Jade T. T. Palmer[1], Emily M.G. Hocking[1] and Monica L. Gerth[1]*

[1]School of Biological Sciences, Victoria University of Wellington, Wellington 6012, Aotearoa, New Zealand.

*Correspondence should be addressed to monica.gerth@vuw.ac.nz

Supplementary Figure 1

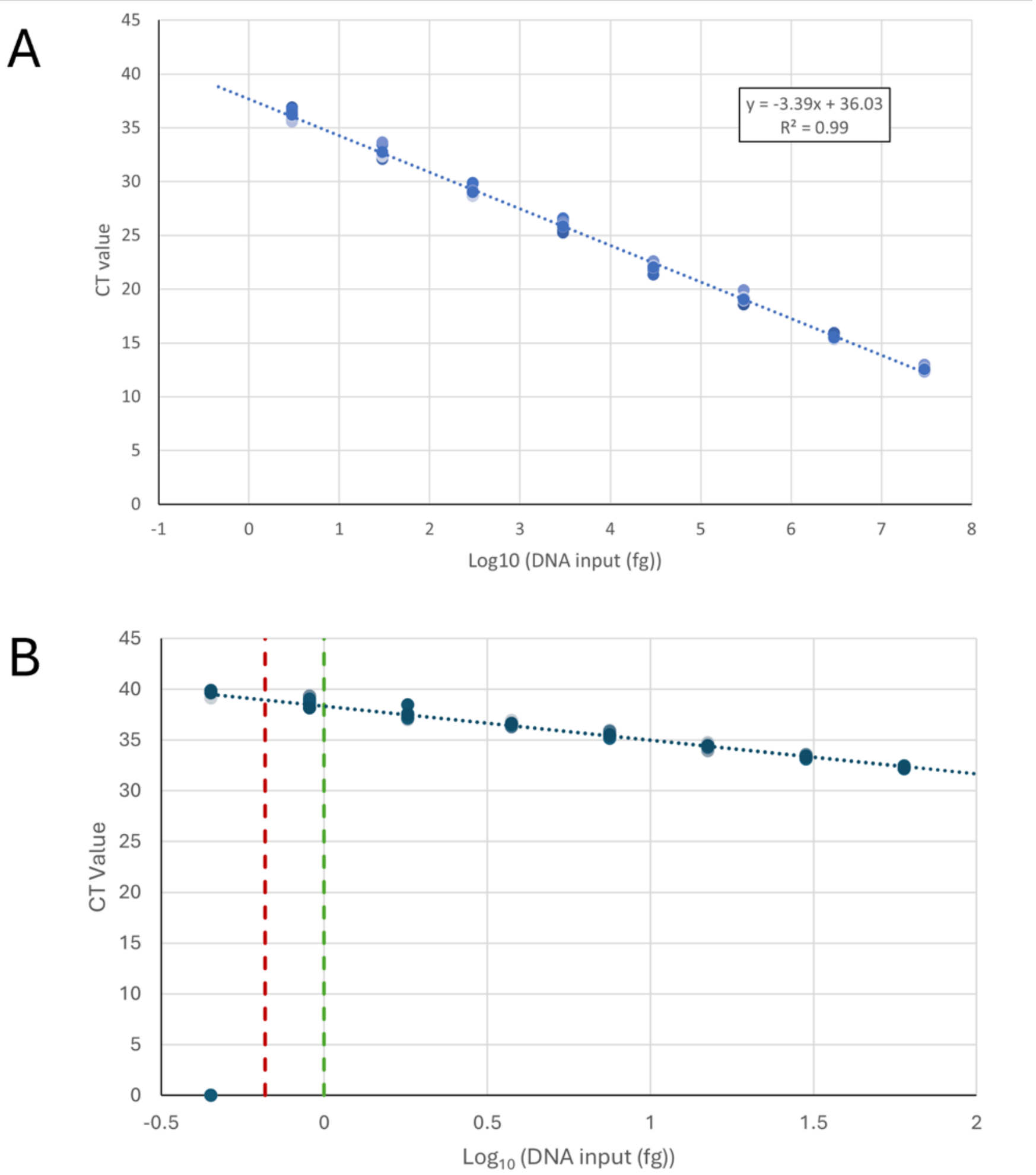

Supplementary Figure 1. Performance and sensitivity of the *Phytophthora agathidicida* (PA) qPCR assay. (A) Standard curve and amplification efficiency. qPCR standard curve generated using PA genomic DNA. Ten standards (ranging from 0.03 fg to 30,000,000 fg (30 ng) were measured using nine replicates per concentration. The lowest standard (0.03 fg) was not detected, and the second-lowest (0.3 fg) was detected in only two replicates and therefore excluded from curve fitting. Using the PA-LTR primers and the PA-LTR-probe, the assay exhibited an amplification efficiency of 97%. The upper limit of quantification was determined to be 30 ng, and the most linear range was 3,000,000 fg (3 ng) down to 3 fg. **(B) Limit of detection (LoD) and limit of quantitation (LoQ).** To precisely define assay sensitivity, nine replicates of seven gDNA standards in twofold dilutions from 0. 16 fg to 60 fg were tested. LoD and LoQ values were calculated using algorithms optimised for eDNA assays (1). The coefficient of variation (CV) threshold for the LoQ was set to 0.35, and the ‘Best’ model settings were applied, as recommended. The LoD (red dashed line) was determined to be 0.7 fg, and the LoQ (green dashed line) was determined to be 1 fg. These thresholds delineate the technical limits for detecting and quantifying PA in environmental DNA extracts, distinguishing true low-level detections from stochastic amplification near the assay's limit.

Supplementary Table 1. Vertical distribution of *Phytophthora agathidicida* (PA) DNA in soil adjacent to eight PA-positive kauri trees. Values represent the mean concentration per depth interval ± standard error. Statistical analysis via One-Way ANOVA indicated no significant difference between depths; $p = 0.372$).

Depth	Mean PA DNA (fg/g soil)
0- 5 cm	126 ± 44
6-10 cm	200 ± 101
11-15 cm	148 ± 86
16-20 cm	25 ± 11

Supplementary Table S2. Tree-by-tree analysis of *Phytophthora agathidicida* (PA) DNA concentrations per depth segment. Mean DNA concentrations (± SD) are shown for three qPCR replicates per depth segment. Depth effects were assessed within each tree using one-way ANOVA followed by Tukey's HSD post-hoc test. The assumption of homogeneity of variance was verified using the Brown-Forsythe test prior to performing ANOVA. Trees with only zero values were not analysed. Trees are grouped by symptom status for presentation only.

Non-Symptomatic	Depth segment cm	Mean PA DNA value ± SD	Grouping[1]	Depth Effect (*F*)	*p*-value
KM2	All depths	0	—	—	—
KM3	0-5	5.6 ± 5.0	a	$F_{(3, 8)}$ = 3.823	0.057
	6-10	0	a		
	11-15	0	a		
	16-20	0	a		
KM5	All depths	0	—	—	n/a

Possibly Symptomatic	Depth segment cm	Mean PA DNA value ± SD	Grouping[1]	Depth Effect (*F*)	*p*-value
KM1	All depths	0	—	—	—
KM4	All depths	0	—	—	—
KM7	0-5	94.7 ± 1.8	a	$F_{(3, 8)}$ = 194.6	<0.0001
	6-10	33.3 ±8.2	b		
	11-15	13.3 ± 4.3	c		
	16-20	12.8 ± 1.9	c		
KM9	0-5	75.2 ± 7.5	a	$F_{(3, 8)}$ = 214.1	<0.0001
	6-10	14.0 ± 1.8	b		
	11-15	6.4 ± 1.5	b		
	16-20	7.0 ± 1.0	b		

Symptomatic	Depth segment cm	Mean PA DNA value ± SD	Grouping[1]	Depth Effect (*F*)	*p*-value
KM6	0-5	415.7 ± 31.1	b	$F_{(3, 8)}$ = 593.2	<0.0001
	6-10	784.9 ±40.0	a		
	11-15	72.8± 2.9	c		
	16-20	7.4 ± 4.1	c		
KM8	0-5	119.9 ± 11.3	c	$F_{(3, 8)}$ = 205.8	<0.0001
	6-10	449.4 ± 37.4	b		
	11-15	537.9 ± 37.2	a		
	16-20	93.4± 9.0	c		
KM10	0-5	154.8 ± 4.3	c	$F_{(3, 8)}$ = 952.4	<0.0001
	6-10	279.4 ± 19.7	b		
	11-15	542.1 ± 10.7	a		
	16-20	19.7 ± 10.2	d		
KM11	0-5	25.7 ± 8.7	a	$F_{(3, 8)}$ = 2.679	0.1179
	6-10	36.1 ± 12.2	a		
	11-15	8.0 ± 11.0	a		
	16-20	37.0 ± 22.4	a		
KM12	0-5	119.6 ± 13.8	a	$F_{3, 8)}$ = 157.4	<0.0001
	6-10	0 ± 0	b		
	11-15	0 ± 0	b		
	16-20	17.9 ± 7.8	b		

[1]Means within a row group (per tree) sharing a common letter are not statistically different at alpha = 0.05 according to Tukey's HSD post-hoc test.

Supplementary Table S3. Tree-by-tree analysis of *Phytophthora* spp. DNA concentrations per depth segment. Mean DNA concentrations (± SD) are shown for three qPCR replicates per depth segment. Depth effects were assessed within each tree using one-way ANOVA followed by Tukey's HSD post-hoc test. The assumption of homogeneity of variance was verified using the Brown-Forsythe test prior to performing ANOVA.

Sample ID	Depth segment cm	Mean DNA value ± SD	Grouping[1]	Depth Effect (*F*)	*p*-value
KM1	0-5	0	a	$F_{(3, 8)}$ = 0.8669	0.4969
	6-10	0	a		
	11-15	2.1± 3.6	a		
	16-20	1.1± 1.0	a		
KM2	0-5	647 ± 16	a	$F_{(3, 8)}$ = 1516	<0.0001
	6-10	27 ± 11	b		
	11-15	50 ± 18	b		
	16-20	36 ± 5.3	b		
KM3	0-5	256 ±28	a	$F_{(3, 8)}$ = 113.5	<0.0001
	6-10	291 ± 26	a		
	11-15	17 ± 6.4	c		
	16-20	107 ±16	b		
KM4	0-5	1104 ± 55	a	$F_{(3, 8)}$ = 197.1	<0.0001
	6-10	790 ± 124	b		
	11-15	44 ± 6.6	c		
	16-20	0 ± 0	c		
KM5	0-5	16394 ± 1108	a	$F_{(3, 8)}$ = 449.7	<0.0001
	6-10	8502 ± 440	b		
	11-15	841 ± 190	c		
	16-20	874 ± 122	c		
KM6	0-5	247 ± 11	c	$F_{(3, 8)}$ = 88.94	<0.0001
	6-10	1236 ± 127	a		
	11-15	306± 3	c		
	16-20	807 ± 114	b		
KM7	0-5	183 ± 28	b	$F_{(3, 8)}$ = 84.44	<0.0001
	6-10	759 ± 106	a		
	11-15	244 ± 15	b		
	16-20	112 ± 8	b		
KM8	0-5	2937 ± 380	b	$F_{(3, 8)}$ = 52.26	<0.0001
	6-10	5995 ± 994	a		
	11-15	3446 ± 194	b		
	16-20	481 ± 22	c		
KM9	0-5	111 ± 26	a	$F_{(3, 8)}$ = 52.26	<0.0001
	6-10	6.5 ± 4.6	b		
	11-15	4.5 ± 1.1	b		
	16-20	0 ±0	b		
KM10	0-5	354 ± 28	b	$F_{(3, 8)}$ = 48.40	<0.0001
	6-10	3685 ± 802	a		
	11-15	321 ± 24	b		
	16-20	774 ± 30	b		

continued next page

KM11	0-5<br>6-10<br>11-15<br>16-20	6660 ± 1142<br>574 ± 48<br>135 ± 18<br>168 ± 13	a<br>b<br>b<br>b	$F_{(3, 8)}$ = 93.37	<0.0001
KM12	0-5<br>6-10<br>11-15<br>16-20	276 ± 24<br>208 ± 29<br>46 ± 9.9<br>39 ± 11	a<br>b<br>c<br>c	$F_{(3, 8)}$ = 102.7	<0.0001

Supplementary Table S4. Statistical comparison of *Phytophthora agathidicida* (PA) DNA concentrations in shallow and deep soil samples collected around trees with detectable PA DNA in the surrounding soil. Values are based on three qPCR replicates per sample. Shallow and deep samples were compared using Welch's t-tests to account for unequal variances.

Sample ID	Shallow Mean ± SD	Deep Mean ± SD	Welch's t-statistic	Degrees of freedom	*p*-value
**TR2**	292 ± 37	51.3 ± 9.1	10.9	2.28	**0.0052**
**TR3**	32.2 ± 1.8	13.4 ± 7.0	4.52	2.27	**0.0357**
**TR5**	28.2 ± 4.6	8.6 ± 3.9	5.66	3.91	**0.0052**
**TR7**	283 ± 103	18.0 ± 5.3	4.47	2.01	**0.0462**
**TR8**	807 ± 133	147 ±11	8.59	2.03	**0.0128**

Reference

1. Klymus KE, Merkes CM, Allison MJ, Goldberg CS, Helbing CC, Hunter ME, Jackson CA, Lance RF, Mangan AM, Monroe EM, Piaggio AJ, Stokdyk JP, Wilson CC, Richter CA. 2020. Reporting the limits of detection and quantification for environmental DNA assays. Environmental DNA 2:271–282.
